# Supplementary material for: A cell atlas of the chick retina based on single-cell transcriptomics
Source: eLife. 2021 Jan 4;10:e63907. doi: 10.7554/eLife.63907 (PMC7837701; doi:10.7554/eLife.63907)
Supplement: Transparent reporting form [file elife-63907-transrepform.docx]

***eLife’s* transparent reporting form**

We encourage authors to provide detailed information *within their submission* to facilitate the interpretation and replication of experiments. Authors can upload supporting documentation to indicate the use of appropriate reporting guidelines for health-related research (see [EQUATOR Network](http://www.equator-network.org/%20)), life science research (see the [BioSharing Information Resource](https://biosharing.org/)), or the [ARRIVE guidelines](http://www.plosbiology.org/article/info:doi/10.1371/journal.pbio.1000412) for reporting work involving animal research. Where applicable, authors should refer to any relevant reporting standards documents in this form.

If you have any questions, please consult our Journal Policies and/or contact us: [editorial@elifesciences.org](mailto:editorial@elifesciences.org).

**Sample-size estimation**

- You should state whether an appropriate sample size was computed when the study was being designed
- You should state the statistical method of sample size computation and any required assumptions
- If no explicit power analysis was used, you should describe how you decided what sample (replicate) size (number) to use

Please outline where this information can be found within the submission (e.g., sections or figure legends), or explain why this information doesn’t apply to your submission:

We profiled approximately 50,000 cells, in three groups (E12, E16, E18). Cell numbers are presented in the text accompanying Figure 1. No power analysis was done; cell numbers were based on our prior experience in generating similar data from mammalian retina.

**Replicates**

- You should report how often each experiment was performed
- You should include a definition of biological versus technical replication
- The data obtained should be provided and sufficient information should be provided to indicate the number of independent biological and/or technical replicates
- If you encountered any outliers, you should describe how these were handled
- Criteria for exclusion/inclusion of data should be clearly stated
- High-throughput sequence data should be uploaded before submission, with a private link for reviewers provided (these are available from both GEO and ArrayExpress)

Please outline where this information can be found within the submission (e.g., sections or figure legends), or explain why this information doesn’t apply to your submission:

All the histological results were confirmed on multiple sections, and representative images are shown in Figures. For high-throughput sequencing, data have been deposited in GEO and the Broad Single Cell Data Portal; the accession numbers can be found in “Data availability” in METHODS. We provide a private link for the reviewers in a separate document.

**Statistical reporting**

- Statistical analysis methods should be described and justified
- Raw data should be presented in figures whenever informative to do so (typically when N per group is less than 10)
- For each experiment, you should identify the statistical tests used, exact values of N, definitions of center, methods of multiple test correction, and dispersion and precision measures (e.g., mean, median, SD, SEM, confidence intervals; and, for the major substantive results, a measure of effect size (e.g., Pearson's r, Cohen's d)
- Report exact p-values wherever possible alongside the summary statistics and 95% confidence intervals. These should be reported for all key questions and not only when the p-value is less than 0.05.

Please outline where this information can be found within the submission (e.g., sections or figure legends), or explain why this information doesn’t apply to your submission:

DE tests were performed using R package “MAST.” This and other metrics for analysis of scRNAseq data are described in “Analysis of scRNAseq data” under METHODs.

In RESULTs, most scRNAseq expression data are presented as dot plots, which show the fraction of cells within a cluster that expressed a particular gene, as well as the expression level. This is stated in Figure 3 legend: “In this and subsequent figures, dot size indicates the proportion of cells that express each gene, and color indicates expression level normalized to its max value among clusters.” All raw data are available at GEO (see above).

Histological data are shown as representative examples. In one case, cell positions are quantified – Figure 5 supplement 1P. Here, the graph shown sample minimum, the lower quartile, the median, the upper quartile, and the sample maximum

(For large datasets, or papers with a very large number of statistical tests, you may upload a single table file with tests, Ns, etc., with reference to sections in the manuscript.)

**Group allocation**

- Indicate how samples were allocated into experimental groups (in the case of clinical studies, please specify allocation to treatment method); if randomization was used, please also state if restricted randomization was applied
- Indicate if masking was used during group allocation, data collection and/or data analysis

Please outline where this information can be found within the submission (e.g., sections or figure legends), or explain why this information doesn’t apply to your submission:

N/A

**Additional data files (“source data”)**

- We encourage you to upload relevant additional data files, such as numerical data that are represented as a graph in a figure, or as a summary table
- Where provided, these should be in the most useful format, and they can be uploaded as “Source data” files linked to a main figure or table
- Include model definition files including the full list of parameters used
- Include code used for data analysis (e.g., R, MatLab)
- Avoid stating that data files are “available upon request”

Please indicate the figures or tables for which source data files have been provided:

All relevant data on gene expression and cell numbers are available through the Single Cell Portal (see above).
